# Exploration of the molecular biological mechanisms and review of postoperative radiotherapy cases in tenosynovial giant cell tumors

**DOI:** 10.3389/fonc.2024.1331815

**Published:** 2024-03-14

**Authors:** Tianwei Zhang, Bin Zeng, Ke Liu, Qin Zeng, Na Wang, Ling Peng, Hongbo Qiu, Xiaomei Chen, Lin Wang

**Affiliations:** ^1^ Department of Oncology, Zigong First People’s Hospital, Zigong Medical Science Academy, Zigong, Sichuan, China; ^2^ Department of Oncology, West China (Airport) Hospital, Sichuan University, Chengdu, Sichuan, China

**Keywords:** tendon sheath giant cell tumor, radiotherapy, molecular mechanism, bioinformatics, benign tumor, benign tumor

## Abstract

Tendon Sheath Giant Cell Tumor (TGCT) is a benign tumor that primarily grows within joints and bursae. However, it has a high postoperative recurrence rate, ranging from 15% to 45%. Although radiotherapy may reduce this recurrence rate, its applicability as a standard treatment is still controversial. Furthermore, the pathogenic mechanisms of TGCT are not clear, which limits the development of effective treatment methods. The unpredictable growth and high recurrence rate of TGCT adds to the challenges of disease management. Currently, our understanding of TGCT mainly depends on pathological slice analysis due to a lack of stable cell models. In this study, we first reviewed the medical records of two female TGCT patients who had undergone radiotherapy. Then, by combining bioinformatics and machine learning, we interpreted the pathogenesis of TGCT and its associations with other diseases from multiple perspectives. Based on a deep analysis of the case data, we provided empirical support for postoperative radiotherapy in TGCT patients. Additionally, our further analysis revealed the signaling pathways of differentially expressed genes in TGCT, as well as its potential associations with osteoarthritis and synovial sarcomas.

## Introduction

Tendon sheath giant cell tumor (TGCT) is a benign tumor that primarily grows within joints and bursae, or extends along the tendon sheath (1). This type of tumor originates from synovial cells or mesenchymal cells differentiated into synovial cells (1). TGCT can be classified into two types: localized and diffuse (2). The localized type includes Giant Cell Tumor of the Tendon Sheath (GCTTS) and Localized Pigmented Villonodular Synovitis (PVNS), while the diffuse type includes Conventional Pigmented Villonodular Synovitis and Diffuse Giant Cell Tumor (2). Most GCTTS occur in the small joints of the hands and feet, but they can also affect other joints or non-joint areas (3–5). Although surgical excision is the standard treatment for TGCT (6), the postoperative recurrence rate ranges from 15% to 45% (3, 7).

For benign tumors such as TGCT, surgeons often maintain a cautious attitude towards radiation therapy. Although some literature reports that postoperative radiation therapy can reduce recurrence rates (8, 9), the existing data are not sufficient to support radiation therapy as a standard treatment for TGCT. Published studies are often limited by small sample size, short-term follow-up, and retrospective analysis methods, thus providing limited information (10–14). Furthermore, TGCT patients are typically young and the disease is not life-threatening, and radiation therapy may increase the risk of fibrosis, joint disease, or other sequelae at the irradiated site. Therefore, whether to consider radiation therapy in the absence of other treatment options remains a controversial issue.

Among the recurrence factors of tendon sheath giant cell tumor, inadequate marginal excision of the tumor may be the main reason for the high recurrence rate. Recurrence may lead to patients needing multiple surgeries and can even result in amputation, causing physical and psychological distress for patients. Therefore, timely postoperative radiotherapy can serve as an effective adjuvant treatment method. Past experiences with radiation therapy for benign diseases such as keloids (15), thyroid eye disease (11), and heterotopic ossification (16) suggest that low-dose radiation therapy is a safe, effective, and low-toxicity treatment method (17). Therefore, there is an urgent need for evidence-based guidelines for postoperative radiation therapy of tendon sheath giant cell tumors, including rational indications, radiation dosage, fractionation methods, and the timing of radiation therapy initiation.

This study reports on two recent cases of Tendon Sheath Giant Cell Tumor (TGCT) treated at our institution. Both patients underwent radiation therapy in our department after surgery. Our goal is to provide more empirical data to deepen our understanding of the role and effectiveness of postoperative radiation therapy in the treatment of TGCT. Through a detailed analysis of these cases, we hope to further clarify the advantages and potential risks of postoperative radiation therapy and explore how to use this treatment method most effectively.

Furthermore, the inadequate understanding of the pathogenesis of TGCT has become a significant challenge hindering its effective treatment. Due to the lack of reliable cell models, existing research mainly relies on pathological sections, which undoubtedly restricts our in-depth understanding of the disease. For this reason, we employed bioinformatics techniques in this study to thoroughly analyze the gene expression profile of TGCT and explore its associations with other diseases. We hope to use this approach to gain a deeper understanding of the molecular mechanisms of TGCT. On the other hand, our study also revealed some genes with clear associations with other diseases, providing new clues for researching the potential connections between TGCT, osteoarthritis, and synovial sarcoma.

## Materials and methods

### Data sources

Datasets GSE55235, GSE176133, GSE35956, GSE67311, and GSE42977 were downloaded from the GEO database (**Table 1**). The GSE176133 dataset includes gene expression data from joint synovial tissues of 3 cases of osteoarthritis and 3 cases of pigmented villonodular synovitis. The GSE55235 dataset includes gene expression data from 10 cases of osteoarthritis patients and 10 healthy individuals. The GSE35956 dataset contains gene expression data from 5 cases of osteoporosis patients and 5 healthy individuals. The GSE67311 dataset includes whole blood gene expression data from 67 cases of fibromyalgia patients and 75 control samples. The GSE42977 dataset includes gene expression data from synovial tissues of 5 synovial sarcoma patients and 7 healthy individuals. All data preprocessing included background correction, removal of missing and low expression data, and data without corresponding gene probes (18). The preprocessed data were then normalized. Gene annotation was completed through the Ensembl or NCBI’s RefSeq database. These steps ensured the accuracy and reliability of the data, providing a basis for subsequent analysis.

**Table 1:** Overview of gene expression datasets from the GEO database.

Dataset ID	Study Content	Patient Samples	Control Samples	Data Type
GSE176133	Gene expression in synovial tissue from osteoarthritis and pigmented villonodular synovitis cases	3 osteoarthritis	3 pigmented villonodular synovitis	Gene expression data
GSE55235	Gene expression in osteoarthritis patients and healthy individuals	10 osteoarthritis	10 healthy individuals	Gene expression data
GSE35956	Gene expression in osteoporosis patients and healthy individuals	5 osteoporosis	5 healthy individuals	Gene expression data
GSE67311	Gene expression in blood samples from fibromyalgia patients and control group	67 fibromyalgia	75 control group	Gene expression data
GSE42977	Gene expression in synovial tissue from synovial sarcoma patients and healthy individuals	5 synovial sarcoma	7 healthy individuals	Gene expression data

We conducted a retrospective analysis of two female patients with Tendon Sheath Giant Cell Tumors (TGCT) who received treatment at Zigong First People’s Hospital from May 2022 to September 2023 and underwent radiation therapy after surgery. For detailed information about these two patients, please refer to the results section.

### Selection of differentially expressed genes and protein-protein interaction network

In this study, the limma package in the R program was used to perform a t-test and a Bayesian test on the RNA-seq in the GSE176133 dataset. Differentially expressed genes were defined using a screening criterion of |Log2(Fold Change)|>1 and P<0.05, with negative values representing down-regulation and positive values representing up-regulation. The ggplot2 package was used to create volcano plots for visualization, and the pheatmap package was used to generate heatmaps for visualization.

The STRING online database (https://cn.string-db.org/) was used in this study to predict and visualize the interactions of target proteins, with a minimum required interaction score of 0.7 to ensure the reliability of the interactions. The filtered data was used to construct a protein-protein interaction network, where nodes represent proteins and edges represent interactions between proteins.

### Functional enrichment analysis of differentially expressed genes: GO and KEGG analysis

In this study, we utilized the clusterProfiler package in R to conduct Gene Ontology (GO) analysis and Kyoto Encyclopedia of Genes and Genomes (KEGG) pathway enrichment analysis. These analyses enabled us to understand the roles of genes in biological processes, cellular components, and molecular functions, as well as the positions and functions of DEGs in biological pathways. To visualize the results, we utilized the enrichplot and ggplot2 functions in the clusterProfiler package to create enrichment bar and circle plots, displaying the most significant enrichment categories and the relative abundance and significance of the genes. Additionally, we used the pathview package to generate KEGG pathway diagrams, providing an intuitive view of the positions of DEGs in specific pathways. At all stages of the analysis, we conducted appropriate statistical tests and controlled the False Discovery Rate (FDR) using the Benjamini & Hochberg method to ensure the statistical significance of the results.

### Construction of a weighted gene co-expression network analysis

We performed a weighted gene co-expression network analysis (WGCNA) using the WGCNA package in R (19, 20). First, the raw gene expression data was normalized and missing values were imputed. Then, outliers were excluded through clustering analysis, and a soft threshold was selected to render the network nearly scale-free. Subsequently, we utilized a hierarchical clustering method based on topological overlap measurements to group genes into modules according to similarities in expression patterns. Then, we calculated the Pearson correlation coefficients between each module and clinical features, to assess their relevance. We selected the module with the strongest correlation with clinical features and extracted its genes. Finally, an intersect analysis was conducted on these genes and the previously identified differentially expressed genes, to screen for potential key genes.

### Feature gene calculation and selection

In this study, we pre-selected differentially expressed genes using the Least Absolute Shrinkage and Selection Operator (LASSO) algorithm (21, 22) and the Support Vector Machine (SVM) algorithm (23, 24). LASSO was used for feature selection and regularization, and the optimal λ parameter was determined by 10-fold cross-validation. SVM was used for classification or regression, and we chose the radial basis function as the kernel function, determining parameters C and γ through grid search and cross-validation. We took the intersection of the results pre-selected by LASSO and SVM as the final feature gene set, based on which we built a predictive model. We then plotted the Receiver Operating Characteristic (ROC) curve and evaluated the predictive performance of the model by calculating the Area Under the Curve (AUC). All calculations were performed in the R language environment, using the `glmnet` package for LASSO calculations, the `e1071` package for SVM calculations, and the `pROC` package for ROC curve and AUC calculations.

### Statistical analysis

Statistical analyses were performed using R software version 3.6.1. Count data was represented by the number of cases or percentages, and was analyzed using the χ2 test, or the Fisher’s exact probability method if needed. Quantitative data satisfying a normal distribution was represented by the mean ± standard deviation, and comparisons between groups were made using a t-test. The association strength between risk factor indicators and the scoring system was evaluated by Spearman correlation analysis. We plotted a visualization curve of the regression model and calculated the c-index to validate its effectiveness. A calibration curve was used to compare the differences between the line chart and the ideal model. Differences were considered statistically significant at P<0.05.

## Result

### Case report

#### Case 1

**Figure 1:**
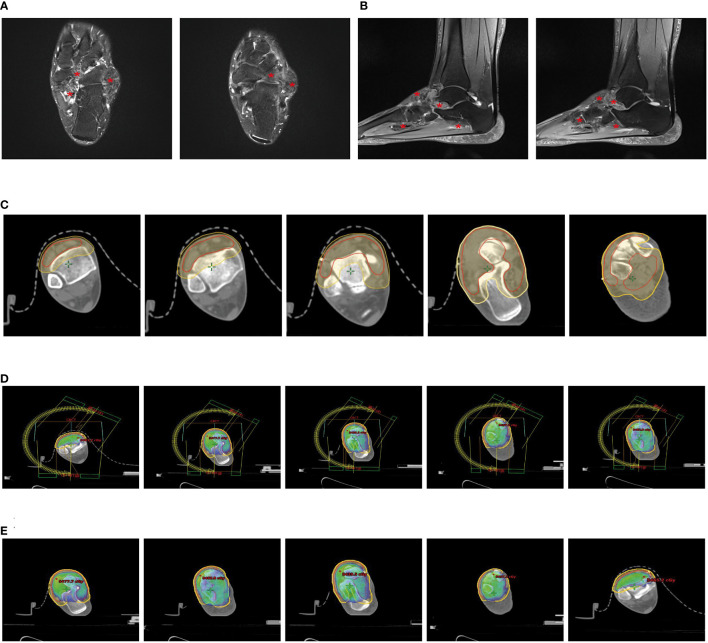
Partial clinical data of a 48-year-old female patient with Tenosynovial Giant Cell Tumor(TGCT). **(A, B)** MRI shows significant thickening of the synovium in the lateral anterior part of the left ankle and the sole of the foot, with the thickened synovium showing villous and nodular changes. **(A)** Transverse section. **(B)** Sagittal section. **(C)** Target area delineation of the patient’s left ankle joint. **(D)** Setting of the irradiation field for the patient’s left ankle joint, which is another critical step in the radiation therapy plan, determining the precision and effectiveness of the radiotherapy. **(E)** Dose distribution in the irradiation field of the patient’s left ankle joint, which helps doctors understand and evaluate the size and distribution of the radiotherapy dose, thus ensuring that patients receive the most effective treatment. # Translation:.

The patient was a 48-year-old female who visited our hospital for a lump in her left ankle accompanied by pain, with a 2-year history of the condition. The initial lump was about the size of a “peanut,” but it gradually enlarged over time and is currently about the size of a “crane’s egg,” causing pain under local pressure. MRI showed significant thickening of the synovium in the anterior external part of the left ankle joint and the foot sole, with the thickened synovium displaying villous and nodular changes. T1w was slightly low signal, the thickened synovium PDW was slightly high signal, the nodules were low signal, and patchy bone erosion was seen at the outer posterior edge of the talus (**Figures 1A, B**).

During the surgery, the patient was placed in the supine position. Under anesthesia and tourniquet control, we first made an incision from the anterolateral side of the left ankle joint. During the surgery, we saw a large amount of villous yellow synovial hyperplasia inside the left ankle joint, 2nd degree damage to the articular cartilage of the tibio-talar joint, and proliferative impact on the talus of the left anterior ankle. Then, we made an incision at the lump on the anterolateral side of the left dorsum of the foot. During the operation, we found a villous yellow tumor about 4.5x4.0x4.0cm in size on the outer side of the left dorsum of the foot, with necrotic tissue in the middle and partial adhesion to the surrounding tissues. Microscopic analysis results showed pigmented villonodular synovitis.

Due to the incomplete resection of the tumor, there was a risk of local recurrence and progression, so we planned to perform local adjuvant radiotherapy on the lesion of the left ankle joint. We used a thermoplastic film to fix the patient’s calf and left ankle joint and collected CT-based images. The clinical target volume (CTV) included the residual tumor and tumor bed area, and a planning target volume (PTV) was generated by extending 7mm from this basis (**Figure 1C**). Our radiotherapy plan aimed to ensure 95% coverage of the PTV (**Figures 1D, E**). According to the treatment plan, we administered a total radiation dose of 48Gy to the tumor in 24 fractions over 5 weeks.

Currently, the patient has completed 15 months of radiotherapy, returned to work, and a telephone follow-up showed that the skin of the patient’s left ankle joint was intact, with local pigmentation, reduced swelling, no pain, and free joint movement, but follow-up imaging data from outside the hospital was not detailed.

#### Case 2

**Figure 2:**
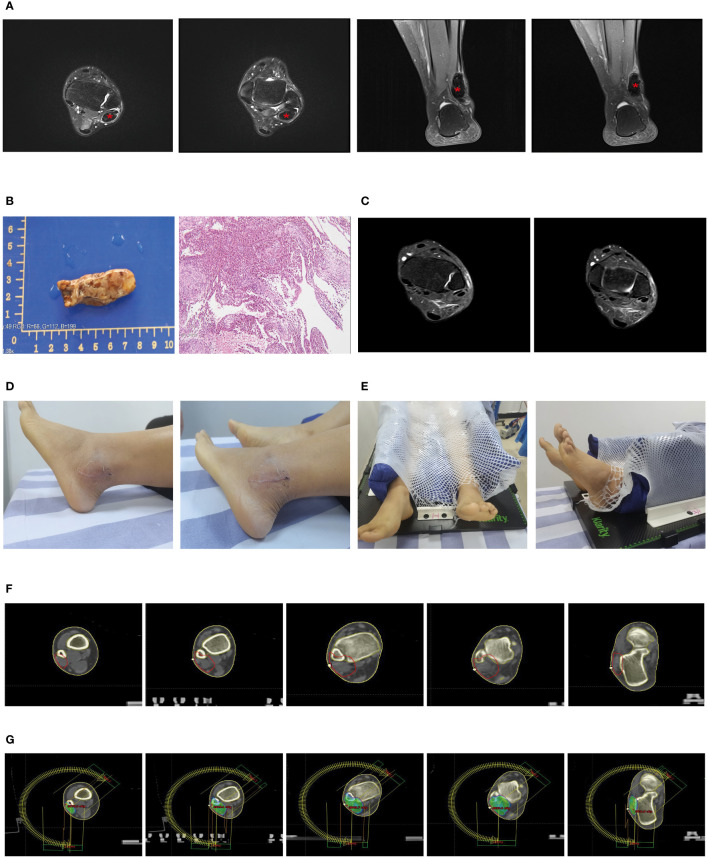
Partial clinical data of a 29-year-old female patient with Tenosynovial Giant Cell Tumor (TGCT). **(A, B)** Preoperative MRI scans of the patient show a mass-like abnormal signal of approximately 4.5cmx1.5cmx1.3cm in the lateral posterior area of the left ankle. The adjacent structures are slightly compressed, and the surrounding soft tissues also exhibit mild swelling. The two images on the left are transverse sections, and the two images on the right are sagittal sections. **(C)** The left image is a macroscopic picture of the patient’s postoperative specimen. The right image is a microscopic picture showing Tenosynovial Giant Cell Tumor with evident nuclear division and pathological mitosis, requiring the exclusion of malignancy. **(D)** One month post-operatively, an MRI showed a few patchy PDw slightly high signals in the left lateral malleolus and a little effusion in the left ankle joint, considered to be postoperative changes. **(E)** The patient’s surgical incision one month after the operation, the wound healed, and there is no obvious swelling locally. **(E)** We used a vacuum pad and thermoplastic film to fix the patient’s calf and left ankle joint to complete the CT-based radiotherapy plan. **(F)** Target area delineation of the patient’s left ankle joint. **(G)** Setting and dose distribution of the irradiation field for the patient’s left ankle joint.

A 29-year-old female patient came to the clinic with over a month’s history of swelling and pain in her left ankle. An MRI scan revealed an irregular mass-like signal about 4.5cmx1.5cmx1.3cm in size in the outer posterior of the left ankle. This signal appeared heterogeneous, predominantly low, with slight compression of the adjacent structures and minor swelling in the surrounding soft tissues (**Figure 2A**).

Under anesthesia and tourniquet control, we incised the skin over the lateral malleolus of the left ankle, revealing a solid tumor approximately 5x3x2cm in size. The tumor wall was intact, the boundary was clear, and there was no adhesion to the surrounding tissue. We successfully removed the entire tumor without damaging the tumor capsule. Microscopic analysis identified the tumor as tenosynovial giant cell tumor, with obvious nuclear division. Pathological mitotic figures were observed, necessitating exclusion of malignancy, a diagnosis agreed upon by remote pathological consultation. Simultaneously, histopathological examination confirmed the absence of tumor at the surgical margins (**Figure 2B**). An MRI one month postoperatively revealed a slight patchy PDW slightly high signal seen in the left lateral malleolus and minor effusion in the left ankle joint, considered postoperative changes (**Figures 2C, D**).

Due to the patient’s recent significant local swelling, active tumor growth, and prominent nuclear division, there was a possibility of malignancy, and we planned to perform local adjuvant radiotherapy on the lesion of the left ankle joint. We used a vacuum cushion and thermoplastic film to immobilize the patient’s calf and left ankle joint (**Figures 2D, E**), and performed CT-based image acquisition. We set the clinical target volume (CTV) as the tumor bed area, and generated the planning target volume (PTV) by expanding 5mm from the CTV (**Figure 2F**). We used Varian Eclipse software to create a volumetric modulated arc therapy plan to ensure at least 95% radiation coverage of the PTV (**Figure 2G**). According to this detailed radiotherapy plan, we administered a total radiation dose of 36Gy to the tumor in 18 fractions over 4 weeks. This volumetric modulated technique allows for more localized radiation, thus protecting the surrounding normal tissue.

To date, the patient has completed a month of radiotherapy and has returned to work. Following up by telephone, we learned that the skin over the patient’s left ankle joint is intact, with slight local pigmentation, and normal function of the left ankle joint.

### Identification and enrichment analysis of differentially expressed genes

Initially, we obtained the GSE176133 dataset from the GEO database, which contains gene expression data from synovial tissues of 3 osteoarthritis cases and 3 pigmented villonodular synovitis cases. We preprocessed and normalized the data using the “limma” package in R.

**Figure 3:**
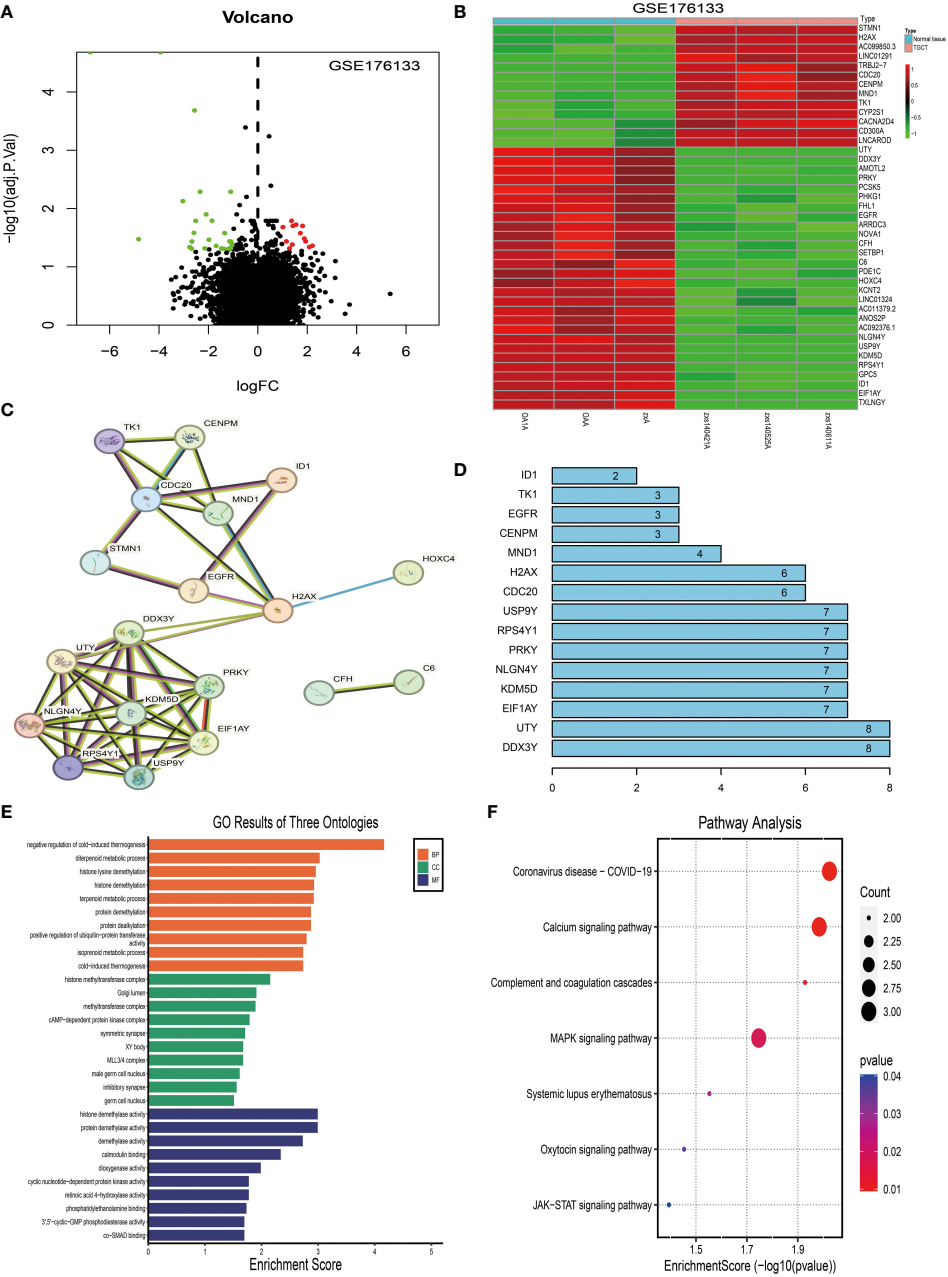
Identification and analysis of differentially expressed genes in tenosynovial giant cell tumor (TGCT). **(A)** Volcano plot analysis of differentially expressed genes in the TGCT GEO-GSE176133 chip dataset. **(B)** Heatmap analysis of differentially expressed genes in the TGCT GEO-GSE176133 chip dataset. **(C)** We predicted potential interactions of proteins encoded by differentially expressed genes using a Protein-Protein Interaction (PPI) network. Figure C displays this network, where each node represents a protein, and each edge represents a protein-protein interaction. **(D)** Connectivity node analysis that we conducted on the predicted protein-protein interaction network. **(E)** Gene Ontology (GO) analysis of the 41 differentially expressed genes. **(F)** Kyoto Encyclopedia of Genes and Genomes (KEGG) analysis of the 41 differentially expressed genes. GO: Gene Ontology; KEGG: Kyoto Encyclopedia of Genes and Genomes.

Subsequently, we compared the differentially expressed genes (DEGs) between the two tissue groups. The results showed that, compared to the synovial tissues of osteoarthritis, there were significant changes in the expression of 41 genes in the synovial tissues of pigmented villonodular synovitis (**Figure 3A**), with 13 genes being upregulated and 28 genes being downregulated (**Figure 3B**).

We then used the STRING website to predict the protein-protein interactions of these 41 DEGs and constructed an interaction network. Network analysis revealed 10 central nodes, including DDX3Y, UTY, EIF1AY, KDM5D, NLGN4Y, PRKY, RPS4Y1, USP9Y, CDC20, and H2AX (**Figure 3C**), with DDX3Y and UTY being particularly central in the network (**Figure 3D**).

Finally, we studied the functions of these DEGs using KEGG (**Figure 3F**) and GO analyses (**Figure 3E**). The results showed that these genes mainly participate in Coronavirus disease - COVID-19, Calcium signaling pathway, Complement and coagulation cascades, MAPK signaling pathway, Systemic lupus erythematosus, Oxytocin signaling pathway, and JAK-STAT signaling pathway (**Figures 3B–E**).

Overall, this study reveals the gene expression differences and potential molecular mechanisms between osteoarthritis and pigmented villonodular synovitis, these findings may have significant implications for the pathogenesis and progression of pigmented villonodular synovitis, providing possible new targets for future diagnostic and therapeutic strategies.

### Analysis of disease correlation between tenosynovial giant cell tumors and osteoarthritis, osteoporosis, fibromyalgia, and synovial sarcoma

In this study, we explored whether there are potential interactions between tenosynovial giant cell tumors and osteoarthritis, osteoporosis, fibromyalgia, and synovial sarcoma. We introduced 41 differentially expressed genes (DEGs) of tenosynovial giant cell tumors into four datasets respectively for differential expression analysis, including osteoarthritis (GSE55235), osteoporosis (GSE35956), fibromyalgia (GSE67311), and synovial sarcoma (GSE42977).

**Figure 4:**
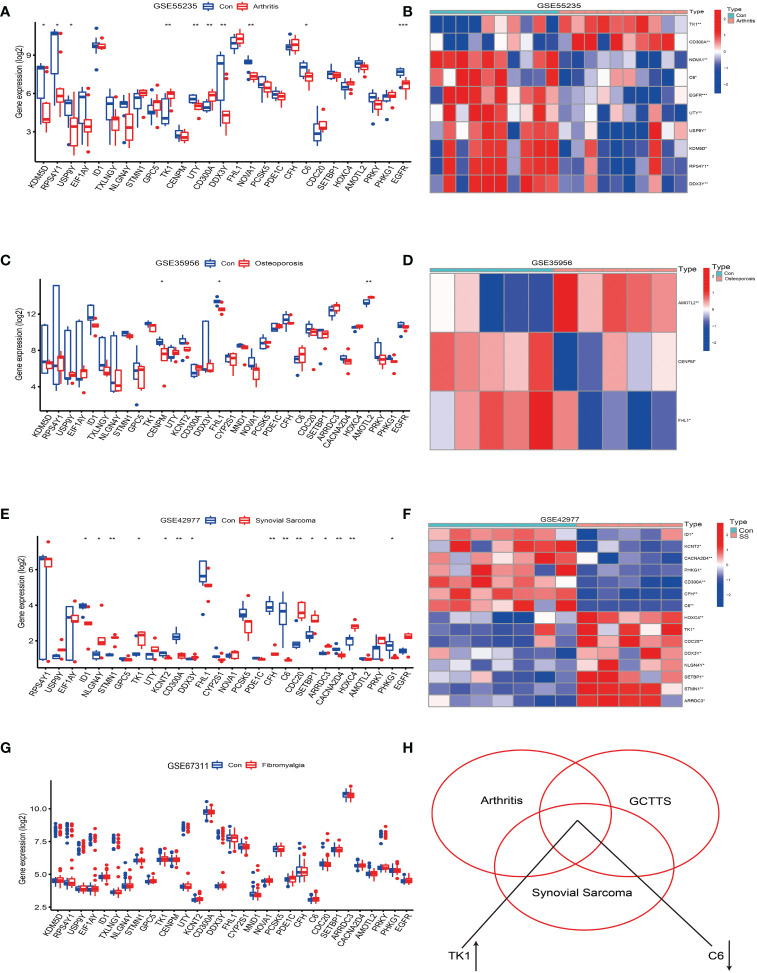
Analysis of the correlation between tenosynovial giant cell tumor (TGCT) and diseases such as osteoarthritis, osteoporosis, fibromyalgia, and synovial sarcoma. **(A, B)** Differential expression analysis of the 41 differentially expressed genes in the Osteoarthritis GSE55235 dataset. **(A)** Bar chart. **(B)** Heatmap of differentially expressed genes. **(C, D)** Differential expression analysis of the 41 differentially expressed genes in the Osteoporosis GSE35956 dataset. **(C)** Bar chart. **(D)** Heatmap of differentially expressed genes. **(E, F)** Differential expression analysis of the 41 differentially expressed genes in the Synovial Sarcoma GSE42977 dataset. **(E)** Bar chart. **(F)** Heatmap of differentially expressed genes. **(G)** Bar chart of differential expression analysis of the 41 differentially expressed genes in the Fibromyalgia GSE67311 dataset. **(H)** Intersection of the 41 differentially expressed genes in the Fibromyalgia GSE67311, Osteoarthritis GSE55235, and Tenosynovial Giant Cell Tumor GSE176133 datasets, revealing an upregulation of TK1 expression and downregulation of C6 gene expression. p<0.001; p<0.01 (**); and p<0.05 (*).

The analysis results showed that compared with normal synovial joints, 10 genes in the arthritis tissue samples exhibited differential expression, accounting for 24%, of which 2 were upregulated and 8 were downregulated (see **Figures 4A, B**). On the other hand, in the osteoporosis tissue samples compared with the healthy population, 3 genes showed differential expression, accounting for 0.7%, of which 1 was upregulated and 2 were downregulated (see **Figures 4C, D**). Notably, we did not find differential expression of these genes in the fibromyalgia tissue samples (see **Figure 4G**). However, compared to the synovial tissue of the healthy population, 15 genes in the synovial sarcoma tissue samples showed differential expression, accounting for 36%, with 8 upregulated and 7 downregulated (see **Figures 4E, F**).

Further analysis revealed that the TK1 gene was upregulated in osteoarthritis, tenosynovial giant cell tumors, and synovial sarcoma, while the C6 gene was downregulated in these three diseases (see **Figure 4H**). Therefore, this study reveals potential shared molecular mechanisms between tenosynovial giant cell tumors and osteoarthritis, synovial sarcoma, particularly the differential expression of TK1 and C6 genes in these diseases may reveal shared pathological processes.

These findings provide a new perspective for deepening our understanding of the molecular mechanisms of these diseases and offer new possibilities for future disease diagnosis and treatment.

### Screening and identification of characteristic genes in osteoarthritis

To further focus on the key pathogenic factors and screen genes with clinical transformation potential, we separately analyzed the 10 differentially expressed genes of osteoarthritis obtained in the aforementioned study.

**Figure 5:**
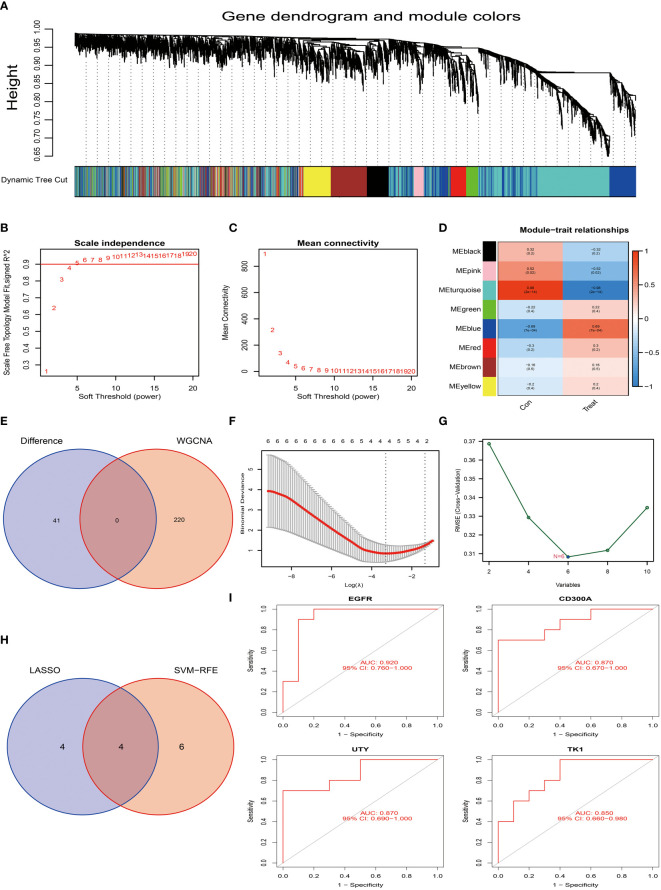
Screening and identification of characteristic genes for osteoarthritis. **(A)** Hierarchical cluster tree and co-expression modules of the gene co-expression network produced based on WGCNA network construction. **(B, C)** Determination of the soft-threshold in WGCNA network construction. **(D)** Analysis of the correlation between each gene co-expression module and osteoarthritis. In the figure, each row represents a gene module, and each column represents a trait. The color shade represents the strength of the correlation between the module and the trait. **(E)** We compared the core module of osteoarthritis with the 41 differentially expressed genes of Tenosynovial Giant Cell Tumor that we previously identified. In this part, we did not find intersecting genes. **(F)** We used the LASSO regression model for feature selection, and through running the LASSO algorithm on our dataset, we identified four candidate feature genes: TK1, CD300A, EGFR, and UTY. **(G)** We used the Support Vector Machine (SVM) method for feature selection, this process generated six candidate feature genes, namely EGFR, NOVA1, CD300A, UTY, DDX3Y, and TK1. **(H)** We compared the feature genes screened out using LASSO and SVM algorithms, and we found that four genes were selected as feature genes in both methods. These genes are TK1, CD300A, EGFR, and UTY, which may play a key role in the development of osteoarthritis. **(I)** To assess the predictive performance of these four candidate feature genes (TK1, CD300A, EGFR, and UTY) for osteoarthritis, we conducted a ROC curve analysis. The ROC curve of each gene was drawn and labeled separately, and we also calculated the AUC (Area Under the ROC Curve) value of the entire model to comprehensively evaluate the predictive performance of the model. p<0.001; p<0.01; and p<0.05.

In this study, we successfully used the GSE55235 dataset and WGCNA network to construct the core genes for osteoarthritis (19, 25, 26). To make the network closer to a scale-free network, we set the soft threshold to 5 and the scale independence to 0.9, thereby obtaining results with relatively high average connectivity, as shown in **Figures 5B, C**.

Based on this setting, we constructed a hierarchical clustering tree and co-expression modules for the WGCNA network, as shown in **Figure 5A**. Among all identified 8 gene modules, the turquoise module contained 220 genes and had the highest correlation with osteoarthritis, with a correlation coefficient of 0.98 (**Figure 5D**). Additionally, the significance of the genes within this module is relatively high, as shown in **Figure 5D**, which further strengthens our findings. However, when we tried to intersect the key genes in the turquoise module with the already identified 10 differentially expressed genes, the result did not yield intersection genes (**Figure 5E**). This may indicate that these differentially expressed genes may be biologically unrelated to the genes in the turquoise module in osteoarthritis.

Next, we employed the LASSO algorithm combined with the Support Vector Machine (SVM) algorithm to screen the characteristics of the 10 differentially expressed genes of osteoarthritis obtained in the above research, hoping to discover key factors with clinical transformation significance. After screening by the LASSO algorithm, we found 4 candidate feature genes, namely TK1, CD300A, EGFR, and UTY (see **Figure 5F**). At the same time, we also applied the SVM algorithm for screening and obtained 6 candidate feature genes, specifically EGFR, NOVA1, CD300A, UTY, DDX3Y, and TK1 (**Figure 5G**). Then, we performed intersection analysis on the genes screened from the two algorithms and finally obtained 4 candidate feature genes, namely TK1, CD300A, EGFR, and UTY (see **Figure 5H**). To further validate the effectiveness of these candidate feature genes, we conducted AUC analyses on them. The results showed that the AUC values of these genes were all greater than 0.85, indicating their high accuracy and feasibility in the diagnosis and prognosis evaluation of osteoarthritis (**Figure 5I**). These findings provide us with important candidate genes and lay the foundation for further functional experiments and clinical research.

### Analysis of the correlation between TK1, CD300A, EGFR, UTY and immune cell infiltration in osteoarthritis

**Figure 6:**
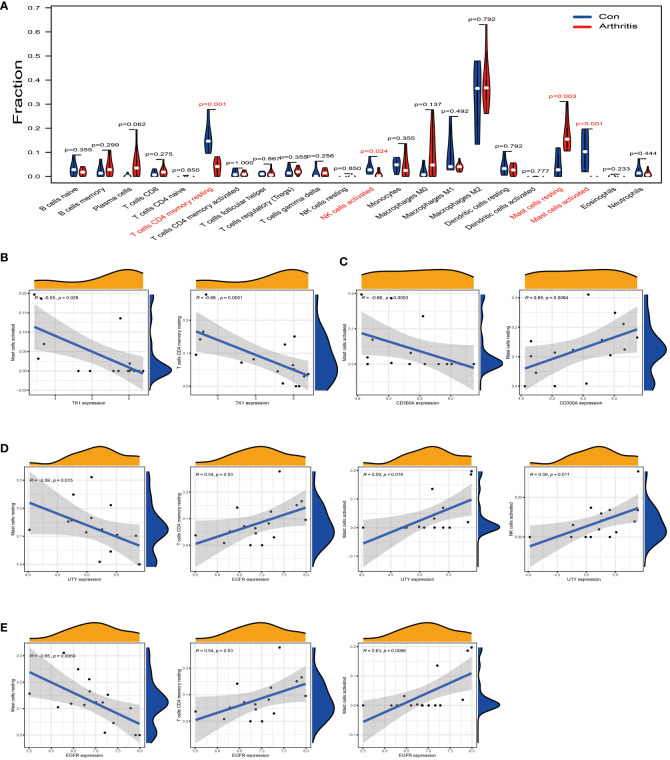
Analysis of the correlation between TK1, CD300A, EGFR, and UTY and the immune cell infiltration in osteoarthritis. **(A)** Difference analysis result of immune cells between normal synovial tissue and osteoarthritis tissue in the GSE55235 dataset. Red represents significantly different types of immune cells. **(B)** In the GSE55235 dataset, we found that TK1 gene expression moderately negatively correlated with the infiltration of T cells CD4 memory resting and Mast cells activated. **(C)** In the GSE55235 dataset, CD300A gene expression moderately negatively correlated with the infiltration of Mast cells activated, but moderately positively correlated with the infiltration of Mast cells resting. **(D)** In the GSE55235 dataset, EGFR gene expression moderately negatively correlated with the infiltration of Mast cells resting, but moderately positively correlated with the infiltration of T cells CD4 memory resting, Mast cells activated, and NK cells activated. **(E)** In the GSE55235 dataset, UTY gene expression moderately negatively correlated with the infiltration of Mast cells resting, but moderately positively correlated with the infiltration of Mast cells activated and NK cells activated. p<0.001; p<0.01; and p<0.05.

We used the GSE55235 dataset to compare immune cell infiltration in normal synovial tissue and osteoarthritis tissue. The results showed that in osteoarthritis tissues, the levels of T cells CD4 memory resting, Mast cells activated, and NK cells activated were significantly downregulated compared to normal synovial membranes (**Figure 6A**, red part). Conversely, the level of Mast cells resting was upregulated in arthritis tissues. These findings suggest that these immune cells may play a role in the pathogenesis of osteoarthritis.

We further conducted a correlation analysis using the same GSE55235 dataset and found that TK1 was moderately negatively correlated with T cells CD4 memory resting and Mast cells activated (R < -0.5) (**Figure 6B**). CD300A was moderately negatively correlated with Mast cells activated (R < -0.5) (as shown in **Figure 6C**, left), but it was moderately positively correlated with Mast cells resting (R > 0.5) (as shown in **Figure 6C**, right). EGFR was moderately negatively correlated with Mast cells resting (R < -0.5) (**Figure 6D**, left), but it was moderately positively correlated with T cells CD4 memory resting, Mast cells activated, and NK cells activated (R > 0.5) (**Figure 6D**, right). Similarly, UTY was moderately negatively correlated with Mast cells resting (R < -0.5) (**Figure 6E**, left), but it was moderately positively correlated with Mast cells activated and NK cells activated (R > 0.5) (**Figure 6E**, right).

In summary, these results provide us with a new perspective to deepen our understanding of immune cell infiltration in osteoarthritis and the possible role of these cells in disease progression. This will help us further research and develop effective treatment strategies for osteoarthritis.

### Screening and identification of characteristic genes in synovial sarcoma

**Figure 7:**
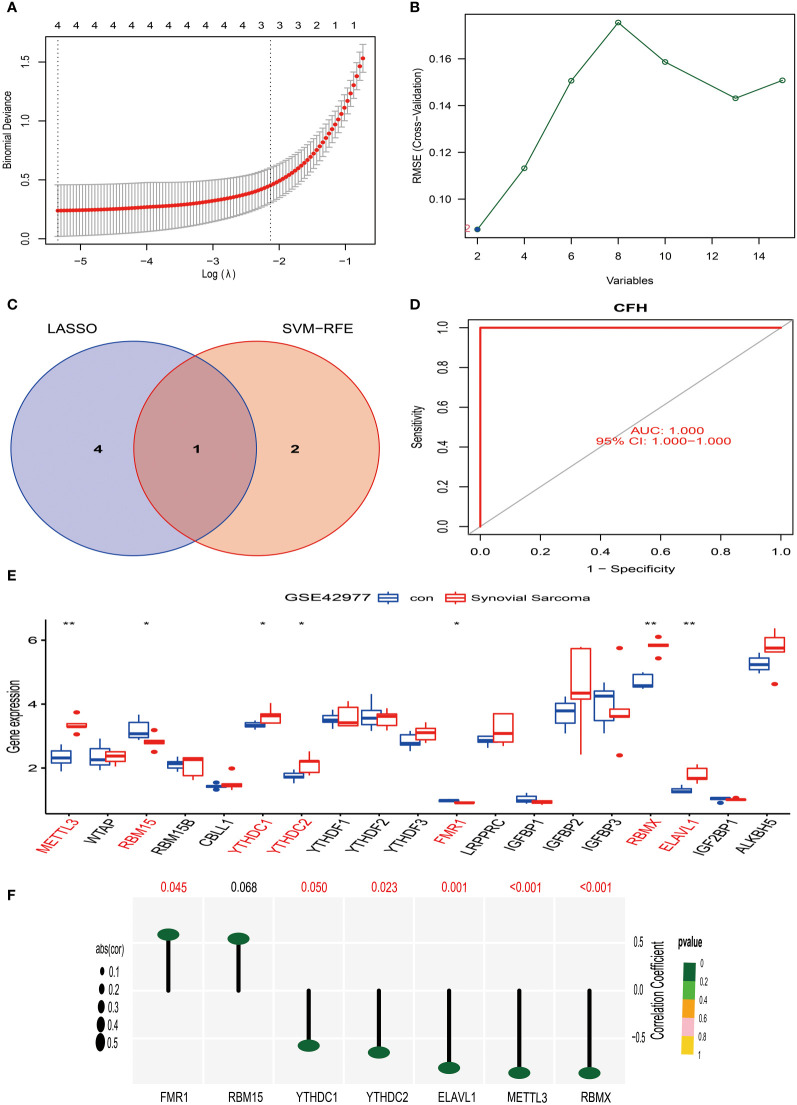
Screening, identification of characteristic genes for synovial sarcoma, and analysis of their correlation with M6A methylation. **(A)** We used the LASSO regression model for feature selection, and through running the LASSO algorithm on the GSE42977 synovial sarcoma dataset, we identified four candidate feature genes: STMN1, CD300A, CFH, and CACNA2D4. **(B)** We used the Support Vector Machine (SVM) method for feature selection, obtaining two candidate feature genes, which are CFH and C6. **(C)** We compared the feature genes screened out using LASSO and SVM algorithms, and we found that CFH was selected as a feature gene in both methods. **(D)** ROC curve analysis. **(E)** In the GSE42977 dataset, the difference analysis result of m6A methylation genes between normal synovial tissue and synovial sarcoma tissue. Red represents significantly differing types of immune cells. **(F)** In the GSE42977 dataset, CFH gene showed a moderate positive correlation with the FMR1 gene, and a moderate negative correlation with METTL3, YTHDC1, YTHDC2, RBMX, and ELAVL1 genes. p<0.001; p<0.01 (**); and p<0.05 (*).

Similarly, we have also applied the LASSO algorithm combined with the Support Vector Machine (SVM) algorithm to screen the characteristics of the 15 differentially expressed genes of synovial sarcoma obtained in the above research using the GSE42977 dataset. The findings of the study showed that the LASSO algorithm selected 4 candidate feature genes, namely STMN1, CD300A, CFH, and CACNA2D4 (**Figure 7A**). Meanwhile, the SVM algorithm screened out 2 candidate feature genes, specifically CFH and C6 (**Figure 7B**). Subsequently, we performed an intersection analysis on the genes screened from the two algorithms, and the result yielded only one candidate feature gene, CFH (**Figure 7C**). This indicates that the CFH gene may play a significant role in synovial sarcoma. To further substantiate this, we conducted an AUC analysis on the CFH gene. The results showed that the AUC value for the CFH gene reached 1, a perfect prediction result, demonstrating the high accuracy of the CFH gene in the diagnosis and prognostic evaluation of synovial sarcoma (**Figure 7D**). This discovery provides a new perspective and potential treatment targets for the research of synovial sarcoma, laying the theoretical foundation for further clinical trials and treatment strategies.

### Analysis of the correlation between CFH gene and m6A methylation in synovial sarcoma

Firstly, we extracted 19 genes related to m6A methylation from the MSigDB website (https://www.gsea-msigdb.org/gsea/index.jsp). Then, using the synovial sarcoma GSE42977 dataset, we compared the m6A methylation genes in normal synovial tissue and synovial sarcoma tissue.

Our research results show that in comparison to normal synovial tissue, the expression of 7 m6A methylation genes differed in synovial sarcoma tissue (**Figure 7E**). Among them, METTL3, YTHDC1, YTHDC2, RBMX, and ELAVL1 were significantly upregulated in synovial sarcoma tissue. This could imply that these genes play a proactive role in the pathogenesis of synovial sarcoma. On the other hand, the expression of RBM15 and FMR1 was significantly downregulated in synovial sarcoma tissue, suggesting they might have an important role in tumor suppression.

To further investigate the correlation of these genes, we performed a correlation analysis on the GSE42977 dataset. The results revealed a moderate positive correlation (R > 0.5) between the CFH gene and the FMR1 gene (**Figure 7F**). However, a moderate negative correlation (R < -0.5) was observed between the CFH gene and the METTL3, YTHDC1, YTHDC2, RBMX, and ELAVL1 genes (**Figure 7F**).

Based on the above, our research provides new clues for discerning the pathogenesis of synovial sarcoma, offering fresh insights for targeted treatment of these genes in the future. However, further experimental validation and in-depth research are required to substantiate these findings.

## Discussion

According to the World Health Organization’s classification of soft tissue and bone tumors in 2020, Tenosynovial Giant Cell Tumor (TGCT) is defined as a locally invasive tumor (27, 28). TGCT mainly impacts young people. Although this disease is usually not life-threatening, it can potentially lead to joint destruction and necessitate repeated surgeries. Hence, its treatment can affect the Quality of Life (QoL) (28). However, there is a lack of prospective data on localized TGCT, with only a few prospective trials for advanced disease. Therefore, most countries lack effective systemic treatment strategies for TGCT (6, 29).

Surgery is the first choice of treatment for most patients receiving initial treatment. However, multiple factors such as incomplete resection during surgery (30), histopathological factors (6, 31), the number and growth pattern of tumors (32), and other influencing factors (33) can lead to the relapse of tenosynovial giant cell tumors, necessitating additional surgeries or even amputation. Early studies administered radiotherapy to patients who underwent incomplete tumor removal or exhibited nuclear fission and bone involvement, with only 2% of high-risk cases relapsing. Besides hyperpigmentation around the scar, no long-term radiation complications were observed (8). Further research indicated that low-dose radiotherapy may not induce sarcomatous changes in GCTTS or joint fibrosis (34).

In our center, for patients with Tenosynovial Giant Cell Tumors (TGCT), we tend to administer aggressive radiotherapy post-surgery, especially for cases occurring in the major joints. This choice is based on the following considerations: First, given the high recurrence rate of TGCT after surgery, radiotherapy can effectively reduce this risk, thereby avoiding additional surgeries (35). Second, our experience indicates that for benign diseases (11, 15, 16), low-dose radiotherapy (20-36Gy) does not cause severe local side effects. Third, TGCT in the major joints usually develops slowly, with larger tumor volumes and more nuclear divisions at diagnosis. Fourth, clinical experience tells us that even if local joint fibrosis changes occur after radiotherapy, the impact on the function of large joints is relatively small. Therefore, compared to repeat surgeries or the use of targeted medications, postoperative radiotherapy is an effective choice. In this study, we provide a detailed demonstration of the target delineation and field design process for radiotherapy of giant cell tumors of the tendon sheath at major joints in our center. We recommend that the clinical target volume (CTV) should include the entire preoperative tumor location. If the tumor invades the joint, the entire joint cavity should be included within the treatment field. The suggested radiotherapy dose should range from 36Gy to 50Gy, and if the tumor has not been completely excised, an increase in the dose to 50Gy should be considered.

The pathogenesis of TGCT is still unclear, and research is constrained by the rarity of cases and a lack of effective cellular models, with basic research remaining at the level of tissue sections (35). Therefore, we employ bioinformatics to study TGCT, a method capable of revealing tumor biological characteristics and gene correlations at the big data level. This approach plays an important role in understanding disease mechanisms, discovering treatment targets, diagnosis, evaluating drug efficacy, and predicting disease prognosis.

In this study, we selected 41 differentially expressed genes in Tenosynovial Giant Cell Tumors (TGCT) through comprehensive gene expression profiling, using a “pick” strategy. We then employed a “circle” strategy for functional clustering and signal pathway analysis. Finally, we identified 10 central proteins within the protein interaction network using a “link” strategy. This “pick,” “circle,” and “link” analysis strategy enabled us to gain a deeper understanding of the molecular mechanisms of TGCT, which is beneficial for guiding clinical treatment.

In the next step, we will compare the gene expression profiles of Tenosynovial Giant Cell Tumors (TGCT) with Osteoarthritis, Osteoporosis, Fibromyalgia, and Synovial Sarcoma, to reveal possible molecular commonalities between these diseases. Our main goal is to uncover potential commonalities at the molecular level among these diseases. By identifying shared biological processes and signaling pathways, we can gain a deeper understanding of the interrelatedness of musculoskeletal diseases. This understanding may help predict related diseases in advance, allowing physicians to take preventive and therapeutic measures, thereby preventing the occurrence of other diseases. In this study, we found molecular sharing between TGCT, Osteoarthritis, and Synovial Sarcoma. This implies that these diseases may have similar pathogenesis or disease backgrounds, which may help us detect and diagnose these diseases earlier, especially Synovial Sarcoma (36), thereby making treatment more timely and effective.

We used deep machine learning algorithms, including the LASSO and SVM algorithms, to identify four candidate feature genes in Osteoarthritis cases: TK1, CD300A, EGFR, and UTY. Further data analysis validated that these genes (TK1, CD300A, EGFR, and UTY) are correlated with the immune microenvironment of Osteoarthritis. However, the roles of TK1, CD300A, and UTY in Osteoarthritis have not yet been reported. Previous research found that mice lacking EGFR activity had fewer surface chondrocytes in their cartilage, reduced lubrication secretion, and weakened mechanical strength of the cartilage surface. These mice showed a significant acceleration of their condition after aging or being affected by Osteoarthritis (37). Therefore, EGFR has become a potential treatment target for Osteoarthritis (38).

In Synovial Sarcoma, we also identified the key gene CFH, although the role of CFH in Synovial Sarcoma has not yet been reported. However, previous research indicates that CFH plays a crucial role in the development and progression of various tumors, such as ovarian cancer (39) and non-small cell lung cancer (40), where changes in CFH expression are closely related to disease severity, prognosis, and survival rate. Our study found that CFH in Synovial Sarcoma is correlated with some genes undergoing m6A methylation. These discoveries emphasize the potential key role that CFH could play in the occurrence and development of Synovial Sarcoma, especially by potentially regulating the expression of tumor-related genes through the process of m6A methylation.

## Conclusion

This study first revisited the case histories of two patients with Tenosynovial Giant Cell Tumors (TGCT). We aimed to explore how to utilize the relevant therapeutic measures most effectively through an in-depth analysis of these cases. Subsequently, using bioinformatics technology, we conducted a comprehensive analysis of the gene expression profile of TGCT, which unveiled its possible pathogenesis as well as potential associations with Osteoarthritis and Synovial Sarcoma. This in-depth exploration could pave the way for improved diagnosis and treatment methods for such conditions.

## Data availability statement

The datasets presented in this study can be found in online repositories. The names of the repository/repositories and accession number(s) can be found below: https://www.ncbi.nlm.nih.gov/geo/.

## Ethics statement

The requirement of ethical approval was waived by Ethics committee member of Zigong First People’s Hospital for the studies on humans because this study is a retrospective analysis, which does not involve human experiment or drug experiment, and will not cause harm to human body, and we have kept patient information confidential. The studies were conducted in accordance with the local legislation and institutional requirements. Written informed consent for participation was not required from the participants or the participants’ legal guardians/next of kin in accordance with the national legislation and institutional requirements. The human samples used in this study were acquired from surgical excision of pathological specimens. Written informed consent was obtained from the individual(s) for the publication of any potentially identifiable images or data included in this article.

## Author contributions

BZ: Conceptualization, Data curation, Formal analysis, Funding acquisition, Writing – original draft, Writing – review & editing. TZ: Writing – original draft, Writing – review & editing. KL: Investigation, Software, Writing – review & editing. QZ: Methodology, Software, Supervision, Writing – review & editing. NW: Data curation, Formal Analysis, Writing – review & editing. LP: Investigation, Methodology, Project administration, Software, Writing – review & editing. HQ: Investigation, Methodology, Writing – review & editing. XC: Formal analysis, Project administration, Validation, Writing – review & editing. LW: Funding acquisition, Methodology, Project administration, Resources, Supervision, Validation, Visualization, Writing – review & editing.
